# Nitric Oxide, a Key Modulator in the Alleviation of Environmental Stress-Mediated Damage in Crop Plants: A Meta-Analysis

**DOI:** 10.3390/plants12112121

**Published:** 2023-05-26

**Authors:** Murtaza Khan, Tiba Nazar Ibrahim Al Azzawi, Sajid Ali, Byung-Wook Yun, Bong-Gyu Mun

**Affiliations:** 1 Department of Horticulture and Life Science, Yeungnam University, Gyeongsan 38541, Republic of Korea; 2 Department of Applied Biosciences, Kyungpook National University, Daegu 41566, Republic of Korea; redflower660@yahoo.com (T.N.I.A.A.);

**Keywords:** nitric oxide, plant responses, biotic stress, abiotic stress, biological processes, redox biology

## Abstract

Nitric oxide (NO) is a small, diatomic, gaseous, free radicle, lipophilic, diffusible, and highly reactive molecule with unique properties that make it a crucial signaling molecule with important physiological, biochemical, and molecular implications for plants under normal and stressful conditions. NO regulates plant growth and developmental processes, such as seed germination, root growth, shoot development, and flowering. It is also a signaling molecule in various plant growth processes, such as cell elongation, differentiation, and proliferation. NO also regulates the expression of genes encoding hormones and signaling molecules associated with plant development. Abiotic stresses induce NO production in plants, which can regulate various biological processes, such as stomatal closure, antioxidant defense, ion homeostasis, and the induction of stress-responsive genes. Moreover, NO can activate plant defense response mechanisms, such as the production of pathogenesis-related proteins, phytohormones, and metabolites against biotic and oxidative stressors. NO can also directly inhibit pathogen growth by damaging their DNA and proteins. Overall, NO exhibits diverse regulatory roles in plant growth, development, and defense responses through complex molecular mechanisms that still require further studies. Understanding NO’s role in plant biology is essential for developing strategies for improved plant growth and stress tolerance in agriculture and environmental management.

## 1. Introduction

As naturally immotile organisms, plants face numerous environmental challenges that affect their growth, development, yield, and survival [1,2]. Abiotic stressors include drought and waterlogging, extreme temperatures, salt stress, and heavy metal (HM) stress. Biotic stressors include herbivores (insects, mites, and mammals) and pathogens (viruses, bacteria, and fungi). Thus, understanding these challenges and developing strategies to mitigate their effects are essential for sustainable agricultural and environmental management [3,4]. Plants have evolved complex mechanisms to respond to and adapt to environmental challenges. For example, under drought conditions, plants can lower the rate of transpiration by closing their stomata to reduce water loss. Plants can also synthesize osmolytes, such as proline and carbohydrates, to maintain water potential in cells and prevent dehydration [5]. To acclimate to excessive salinity stress, plants can control ion transport and accumulation, produce suitable solutes, and alter their metabolism [6]. The production of heat shock proteins, which prevents protein denaturation and maintains membrane integrity, can be induced by plants in response to high temperatures [7]. Plants can produce chelators to bind and sequester toxic metals in response to HM toxicity, and they can also activate antioxidant mechanisms to scavenge reactive oxygen species (ROS) [8]. In response to an herbivore attack, plants increase the accumulation of secondary metabolites, such as alkaloids, terpenoids, and phenolics, that can either repel herbivores or attract their natural enemies [9]. During pathogen attacks, the pattern recognition receptors (PRRs) in plants can activate their immune systems by recognizing pathogen-associated molecular patterns (PAMPs). The synthesis of phytohormones and defense-related metabolites is induced by a series of signaling processes triggered by this identification [10]. Moreover, in response to these environmental challenges, plants produce different signaling molecules, including melatonin (MEL), ROS, and reactive nitrogen species (RNS) [11,12].

RNS are a group of highly reactive molecules, such as NO, that play pivotal roles in plant growth and developmental processes, including seed dormancy and germination, root and shoot growth, vascular differentiation, stomatal movement, nodulation, nutrient uptake, flowering, fruit ripening, as well as growth and development of other vegetative and reproductive tissues [8,13,14,15]. NO has been associated with an improved root system, stomatal movement, hypoxic tolerance, upregulation of gene transcript, antioxidant system, and hormonal regulation during drought and flooding stress response in plants [16,17,18]. During slat stress, NO regulates ion homeostasis, water uptake, antioxidant system, transcript accumulation, and hormone biosynthesis [19]. NO has also been shown to protect plants against extreme temperatures and HMs by regulating the antioxidant system, the expression of stress-related genes, proteins, and hormones [13,20,21], while its significant roles in enhancing the expression of defense-related genes, secondary metabolites, phytohormones, interaction with other signaling molecules, such as ROS, and induction of systemic responses have been shown during herbivore attacks [22]. Similarly, the defense properties of NO in plants against pathogenic infections have been demonstrated by the induction of defense-related genes, such as pathogenesis-related (PR) genes; activation of physiological defense responses, such as ROS production, callose deposition, and hypersensitive response (HR); regulation of plant hormones; enhancement of pathogen-associated molecular pattern-triggered immunity (PTI); effector-triggered immunity (ETI); and systemic acquired resistance (SAR) [23].

Compounds, such as sodium nitroprusside (SNP), S-nitrosocysteine (CySNO), and S-nitrosoglutathione (GSNO) are NO donors [24] and are potentially crucial for the improvement of plant defense system against stressful environmental conditions. Overall, NO is a key determinant of plant adaptation to abiotic and biotic stresses that regulate numerous physiological and biochemical processes, leading to enhanced plant tolerance for sustained growth and survival under stressful environmental conditions [25,26]. Thus, this current review explored the recent advances in NO biosynthesis and its role in plant growth, development, and stress mitigation.

## 2. NO Production

Animals have well characterized systems for controlling NO production and signaling. Despite evidence of its functional presence in plants, the metabolic origin of NO and its role in plants signaling cascades still remains unknown [27]. Numerous NO synthesis mechanisms have been proposed to occur in various probable production sites, including the apoplast, chloroplast, mitochondria, peroxisome, and plasma membrane [26]. Several studies have identified eight distinct enzymatic (oxidative and reductive) and nonenzymatic processes that are involved in NO production [23,26]. NO is synthesized through the oxidative route by oxidizing L-arginine or other polyamines (PAs), as well as hydroxylamines (HAs) [28]. The reductive process mainly depends on nitrate reductase (NR) and other reductive enzymes found in the plasma membrane and mitochondria [29]. The nonenzymatic reduction of nitrite (NO^2−^)/NO in the acidic compartments of plant tissues results in nonenzymatic NO generation [29,30].

### 2.1. Oxidative Pathways of NO Production

Similar to mammalian oxidative processes, plants can produce NO by oxidizing molecules containing nitrogen (N) [31]. NO synthase (NOS) proteins and NOS-producing genes have been found in prokaryotes, unicellular eukaryotes, invertebrates, and vertebrates, including mammals; however, NOS enzymes are yet to be discovered in higher plants [32]. In the leaf peroxisomes and chloroplasts of green algae and vascular plants, L-arginine is oxidized by enzymes to generate citrulline and NO [33]. Nicotinamide adenine dinucleotide phosphate (NADPH) and the absence of Ca^2+^ are necessary for L-arginine oxidation in chloroplasts, whereas NADPH, FAD, FMN, Ca^2+^, and calmodulin are needed for peroxisomal oxidation [34,35].

The oxidation of PAs and HAs in plant cells leads to NO generation [36]; however, the precise mechanism underlying this process is still unknown [37]. A potential pathway involving the interaction between PAs and NR-catalyzed NO [38], as well as a secondary impact of polyamine production on L-arginine metabolism, has been proposed [39]. Although hydroxylamine, a byproduct of the nitrification process, can be converted to NO in tobacco cell cultures, the underlying mechanism for this process is still unknown [40], and this process might be used instead of oxidative NO production from L-arginine.

### 2.2. Reductive NO Biosynthesis Pathways

The major substrate for reductive pathways is NO^2−^, which is mediated by NR, NO-forming nitrite reductase (NOFNiR), and mitochondrial nitrite reduction (MNR) [41]. The reduction of NO^2−^ to NO by NR is pivotal during stressful conditions in cyanobacterium, green algae, and vascular plants [32,42]. The key and oldest enzymatic source in plants is thought to be NO synthesis from NO^2−^ through NR [41]. This pathway produces low amounts of NO under normal circumstances because the major substrate for NR is nitrate (NO^3−^), and NO^2−^ reduction accounts for only 1% of the NR activity. The primary sites for NR activity are the cytoplasm and chloroplast [43]. NO^2−^/NO-reductase (NI-NOR) can also convert NO^2−^ to NO by using reduced cytochrome c as an electron acceptor [44]. Moreover, when NO^2^ is used as an alternative electron acceptor during ATP synthesis in the mitochondrial complexes III and IV, NO^2−^ is reduced to NO [43], causing plant cells to become hypoxic and mitochondria to produce NO, which inhibits the activity of cytochrome c oxidase to improve the energy status of hypoxic cells [41]. Hemoglobin in the cytosol of hypoxic cells oxidizes NO released by the mitochondria, resulting in a constant supply of NO_2_ for ATP synthesis [45]. NO^2−^ reduction in plant peroxisomes under hypoxic or anoxic environments is an alternative route for producing reductive NO. Plant PM, cytosol, and endoplasmic reticulum generate reductional NO in a similar manner [46].

### 2.3. Nonenzymatic NO Synthesis Pathways

Nonenzymatic NO generation is triggered by the release of NO from nitrous acid. This necessitates a low pH due to gibberellin (GA) and abscisic acid (ABA), resulting in an acidic apoplast [47]. Nonenzymatic conversion of NO^2−^ to NO mediated by phenolic chemicals was observed in the apoplast of the barley aleurone layer [48]. Moreover, NO is used to break up seed dormancy, indicating that both NO^2−^ and enzymatic NO production are necessary for healthy seed germination [49]. The release of NO from nitrosoglutathione (GSNO), which is yet to be fully investigated, is another potential route for nonenzymatic NO generation [50].

## 3. Role of NO in Plant Growth and Development

**Figure 1:**
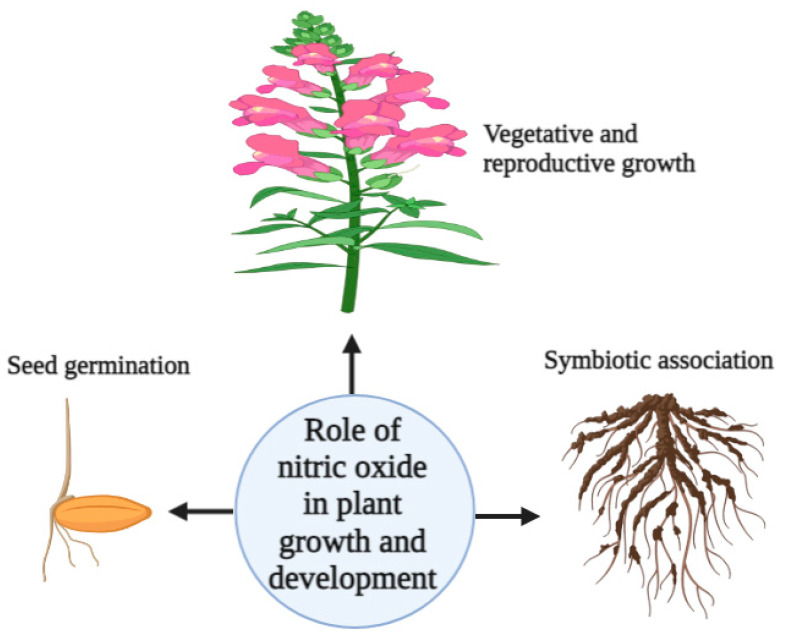
Schematic diagram showing the role of nitric oxide in plant growth and development. The figure was generated by BioRender.com.

NO plays a pivotal role in the regulation of plant growth and development by significantly enhancing seed germination through the breakdown of storage reserves and the regulation of target germination genes, proteins, and hormones, such as ABA [51]. SNP application has been shown to increase seed germination of wheat [52]. In addition to promoting seed germination, NO is crucial for seed fatty acid composition and oil accumulation [53]. NO can also improve the growth and development of roots and shoots by enhancing cell division, elongation, and differentiation. NO can interact with various phytohormones, such as auxins (AUX), GA, and cytokinins (CK), to regulate plant growth and development. For example, NO was shown to not only enhance the production and transport of AUXs, which are necessary for root and shoot growth, but also to interact with GA to promote stem elongation and seed germination [23,41]. Moreover, NO could significantly improve plant tolerance to various abiotic stress conditions, such as drought, flooding, extreme temperature, salinity, and HM stresses, as well as biotic stresses, by regulating the expression of stress-related genes and inducing the production of defensive secondary metabolites [13,17,26,54]. NO also establishes the symbiotic association between plants and beneficial microbes to enhance plant growth and development [23]. However, at higher concentrations, NO negatively affects chlorophyll and photosynthesis [55]. Overall, NO is a key regulator of plant growth and development, and its effects are mediated by interactions with various signaling pathways and plant hormones. By modulating these pathways, NO helps plants optimize their growth and development to adapt to changing environmental conditions. The role of NO in seed germination, enhancement of symbiotic associations, and improvement of vegetative and reproductive growth is shown in Figure 1.

## 4. Role of NO in Plant Abiotic Stress Response

Abiotic stresses are nonliving factors that can adversely affect plant growth and development, leading to decreased productivity. Understanding the mechanisms of plant response to these stresses is crucial for developing strategies for improved plant adaptation and increased productivity. NO is an important signaling molecule that can induce physiological, molecular, and biochemical changes in plants under different abiotic stresses, such as drought, flooding, extreme temperatures, salinity, and HM stresses [56,57]. During the abiotic stress response, NO significantly enhances plant growth and development by activating stress-related genes, proteins, hormones, and the antioxidant system [26]. The role of NO in mitigating different abiotic stresses is discussed in the following sections.

### 4.1. Drought and Flooding Stress

Drought is a serious abiotic plant stressor that results from prolonged exposure to low water availability in the soil, which is caused by various factors, such as low rainfall, high temperatures, or water loss through transpiration [3,5]. Drought stress adversely affects plant growth and development, leading to loss in yield. Developing strategies to improve plant tolerance to drought stress is vital for maintaining crop productivity and ensuring food security in regions with low water availability [58]. Both endogenous and exogenous NO can significantly enhance plant growth and development under abiotic stress conditions [59,60]. NO has been shown to regulate stomatal movement during drought conditions by activating calcium ion channels, which leads to stomatal closure and reduced water loss through transpiration [25]. Drought stress can increase the accumulation of ROS in plant cells, which damages cellular components, such as proteins and lipids. Studies have shown that NO activates antioxidant enzymes, such as superoxide dismutase (SOD) and catalase (CAT), which scavenge ROS to mitigate oxidative damage [61]. Similarly, NO was shown to regulate the expression of drought related genes, such as *AO3* and *NCED3*, to enhance the *Arabidopsis thaliana* defense system against drought stress, and these genes could also regulate ABA production, stomatal closure, and *A. thaliana* acclimation to drought stress [16,17]. Moreover, NO has been reported to induce the accumulation of compatible solutes in plants, such as proline, which can maintain cellular water balance and protect cellular structures during drought stress [59]. In addition, NO could significantly reduce the production of hydrogen peroxide (H_2_O_2_), superoxide anion (SOA), and malondialdehyde (MDA) in plants during drought stress [62]. The NO activity is concentration dependent; for example, during drought stress, the recovery effects of 100 µM SNP were more effective than 50 µM [63]. NO donors significantly improve growth and development in different plants under drought stress [54,63]. In summary, the application of NO donors can enhance drought stress tolerance in plants by regulating physiological processes associated with water uptake, photosynthesis, and oxidative responses. The effectiveness of NO donors can vary depending on plant species and the severity of drought stress. Further studies are still needed to develop strategies for optimizing the application of NO donors in crop production systems under drought stress conditions.

Flooding stress occurs when plants are subjected to excessive water in the soil or around the root zone; it can severely impact growth and productivity due to reduced oxygen availability, nutrient deficiencies, and other adverse physiological changes. The effects of flooding stress on plants can vary depending on its severity and duration [64,65]. Endogenous or exogenous NO has been shown to significantly improve plant tolerance to flooding stress [66]. For example, functional evaluation of NO donors, such as SNP and CySNO, at the physical, biochemical, and molecular levels in soybean during flooding stress revealed their capacity to significantly enhance plant growth and development, antioxidant responses, ABA production, and upregulated expression of NO synthesis-related genes, such as *NR* and *S-nitrosoglutathione reductase* (*GSNOR1*) [67]. Similarly, application of NO donors was shown to significantly improve the flooding stress tolerance of soybean, cotton, and maize crops [68,69,70], which demonstrate their potential as active substances for mitigating the adverse effects of flooding stress.

### 4.2. Extreme Temperature Stress

Extreme temperature and heat stress occurs when plants are exposed to either too high or low temperatures outside their normal range, which can adversely alter their physiological, molecular, and biochemical processes and affect their growth and development, as well as productivity [71,72]. Previous studies have shown that endogenous or exogenous NO can significantly improve plant thermotolerance; for example, NO production was demonstrated to significantly enhance the thermotolerance of *Vicia faba* plants, while the application of an NO scavenger, 2-4-carboxyphenyl-4,4,5,5-tetramethylimidazoline-1-oxyl-3-oxide (cPTIO) could significantly reduce NO production, which also demonstrated that NO is endogenously produced in plants during heat stress [20]. Thus, NO donors could be sued to improve plant tolerance at high temperatures. For example, increased production of oxidative stress markers, such as H_2_O_2_ content in wheat during high temperature stress, were significantly decreased by application of NO donors [73]. Similarly, the application of SNP showed significantly improved heat-stress-tolerance of *Lablab purpureus* L. plants [74], while its application could also reduce the adverse effects of heat stress on rice plants [75]. Overall, NO plays critical roles in the regulation of plant responses to heat stress and enhancing its levels in plants has been shown to improve heat stress tolerance, which makes it a potential target for developing strategies for mitigating the negative effects of heat stress on plant growth and productivity.

Chilling stress occurs when plants are exposed to temperatures below their optimal range, typically between 0 °C and 15 °C. The effects of chilling stress on plants can vary depending on the severity and duration of the stress, plant species, and plant developmental stages [76]. NO has been reported to play a positive role in alleviating the negative effects of chilling stress on plant growth and development [77] by regulating the transcript accumulation of stress response genes, such as those encoding for antioxidant enzymes, osmolyte biosynthesis, and stress signaling pathways [78]. NO can activate the antioxidant defense system by upregulating the activity of antioxidant enzymes, such as SOD and CAT, to eliminate ROS mediated oxidative damage of plant cells during chilling stress, while the application of cPTIO could reduce the positive effects of NO [79]. NO was also reported to modulate ion transport across plant membranes, particularly the plasma membrane and tonoplast, to maintain ion homeostasis, which can be disrupted by chilling stress, leading to altered plant growth and development [76]. Chilling stress can significantly reduce photosynthesis in plants, leading to defects in plant growth and development, and NO has been shown to enhance photosynthesis by improving the efficiency of the photosynthetic apparatus and by regulating the expression of photosynthetic genes [80]. In addition, NO has been shown to mitigate the adverse effects of chilling stress on tomato, cucumber, and rice plants [76,78,81]. Interestingly, the application of an NO donor was also demonstrated to improve the quality of zucchini fruits during storage at 4 °C [82]. Similarly, the application of NO donors enhanced banana fruit resistance to low temperatures, which also induced the production of endogenous NO [83]. These findings suggest that NO donors can be used to enhance plant tolerance to extreme temperature stress.

### 4.3. Salinity Stress

Salinity is a major abiotic stress that affects plant growth and productivity worldwide. High salt concentrations in the soil can cause osmotic imbalance, ion toxicity, and oxidative damage to plant cells, leading to decreased photosynthesis, growth inhibition, and ultimately plant death [6]. However, NO has been shown to regulate the transport and accumulation of ions, such as sodium ion and potassium ions, in plant cells during salinity stress by modulating the activity of ion channels and transporters [84,85]. Overproduction of ROS in plant cells and oxidative damage induced by salinity stress can be alleviated by NO, which is a signaling molecule that can induce antioxidant enzymes, such as SOD and CAT, to scavenge ROS directly [86]. Salinity stress-induced changes in plant hormones balance, such as ABA and CK, which affect plant growth and development, can be maintained by NO through modulation of hormone signaling pathways, leading to improved plant salinity stress tolerance [87]. Moreover, NO could activate the expression of salt stress-related genes, such as *HIPP38*, *GR1*, and *P5CS2* in rice [86]. Application of NO donors has been reported to significantly enhance salt tolerance in different plant species, such as *Salicornia persica*, *Vigna radiata*, *Crocus sativus*, and *Triticum aestivum* [88,89,90,91]. Overall, NO mitigates salinity stress by maintaining ion homeostasis, protection against oxidative stress, modulating hormone signaling, and activating stress responsive genes to improve plant acclimation to salinity stress.

### 4.4. HM Stress

**Figure 2:**
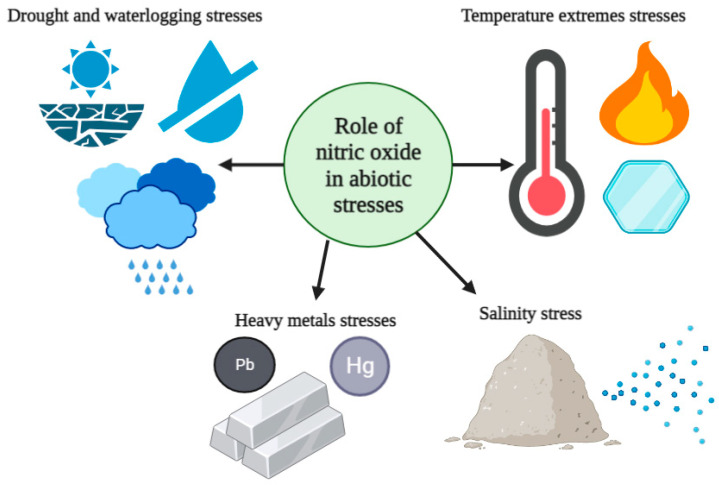
The roles of nitric oxide in plant abiotic stress responses. The figure was generated using BioRender.com.

The increased use of fertilizers in agriculture, industrialization, anthropogenic activities, and improper waste management have exacerbated HM contamination in the soil, posing major issues for agriculture worldwide [92]. High-concentration intake of HMs, such as aluminum (Al), arsenic (As), cadmium (Cd), chromium (Cr), cobalt (Co), copper (Cu), mercury (Hg), nickel (Ni), and lead (Pb), can impair physiological, biochemical, and molecular plant processes [13,93]. Several studies have demonstrated that exogenous application of NO donors, such as SNP, can enhance plant tolerance to HM stress by increasing growth and chlorophyll contents in rice plants exposed to mercury (Hg), chromium (Cr), copper (Cu), Zinc (Zn), and lead (Pb) stress [8,13]. SNP application was also shown to mitigate the toxic effects of Hg, Cd, and Pb stress in other plants, such as soybean, *Satureja hortensis*, *Pimpinella* anisum, and rice plants [8,94,95]. Moreover, the application of NO donors increased Cd, As, and Cu stress tolerance in peanuts, *Brassica juncea*, and tobacco [96,97,98]. The protective effects of NO against HM stress could be attributed to several mechanisms, including regulation of the expression of genes involved in metal detoxification and the antioxidant system [13,99]; scavenging of ROS produced by HM stress, thereby preventing oxidative damage [100]; and enhancing the production of phytohormones, such as ABA and jasmonic acid (JA), which are involved in plant stress responses [101]. Despite studies showing that both endogenous and exogenous NO are crucial for mitigating the effects of HM stress, NO-mediated alleviation of HM stress differs depending on the NO donor, HM concentration, duration of exposure, plant species, and tissue exposed [102]. In summary, NO enhances HM stress tolerance in plants through complex mechanisms, and its ability to regulate gene expression, scavenge ROS, and enhance hormone production makes it a promising target for improving plant tolerance to HM stress. The roles of NO in mitigating abiotic stresses, such as drought, waterlogging, extreme temperatures, salinity, and HM stress, are illustrated in Figure 2.

### 4.5. A Summary of the Role of NO in the Mitigation of Abiotic Stresses

Abiotic stresses, such as drought, salinity, extreme temperatures, and HMs, can disrupt normal plant growth and development, leading to reduced crop yields and economic losses. As a signaling molecule, NO plays an important role in the regulation of plant growth and development, as well as in the response of plants to environmental stressors. NO has been reported to regulate the expression of genes involved in stress responses, such as those encoding antioxidant enzymes, osmolyte biosynthesis enzymes, ABA, and stress-responsive transcription factors. NO has been shown to induce the ABA pathway and drought stress-related genes, such as *AO3*, *NCED3*, and *bZIP*, thereby significantly enhancing drought stress tolerance in *A. thaliana* [16,17]. Similarly, SNP application significantly enhanced the expression of HM stress-related genes, such as *PCS1*, *PCS2*, *MTP1*, and *MTP5*, in rice plants under HM stress [13]. Oxidative damage to cellular components by abiotic stress-induced ROS overproduction can be alleviated by NO due to its antioxidant properties or ability to induce antioxidant enzymes activities [103]. During response to drought or salinity stress, NO has been reported to induce the accumulation of compatible solutes, such as proline to enable osmotic adjustments in plants [59,104]. Under conditions of water scarcity or high temperatures, plants may close their stomata to preserve water and reduce transpiration. NO has been reported to modulate stomatal closure, allowing plants to maintain a balance between water conservation and photosynthetic efficiency [17,105]. Moreover, increased production of oxidative stress markers, such as H_2_O_2_, SOA, MDA, and EL, during abiotic stresses can be reduced by the application of NO, which activates the antioxidant system [76,89,106]. The roles of NO in the mitigation of different abiotic stressors are shown in Table 1.

## 5. The Role of NO in Biotic Stress Responses

Biotic stresses are caused by biological factors, such as pests, pathogens, and competing plants, which can have detrimental impacts on plant growth, development, and productivity, leading to significant economic losses in agriculture. Pests such as insects and mites are potential vectors that can damage plant tissues, reduce photosynthesis, and transmit plant viruses. Fungal-, bacterial-, viral-, and nematode-transmitted infections can cause wilting, stunted growth, and decreased yield. Competing plants can outperform crops for resources such as water, nutrients, and light, leading to reduced yields [117].

Plants have evolved complex defense mechanisms to counteract biotic stress. These include physical barriers, such as thorns and trichomes, as well as chemical defenses, such as the production of toxic bioactive compounds and the activation of defense-related genes. However, biotic stressors can often overcome these defenses, leading to significant crop losses [26,118,119]. Efforts to mitigate biotic stress include the use of pesticides, crop genetic engineering, and crop rotation. However, these approaches can have negative environmental impacts, leading to the development of pesticide-resistant pests and diseases. Therefore, sustainable plant protection strategies that consider the complex interactions between plants and biotic stressors are needed [120]. NO has been reported to act as a signaling molecule in response to biotic stressors [26], and its roles in response to insects and pathogens is discussed below.

### 5.1. Role of NO in Plant and Insect Interaction

Insects can cause various negative effects in plants, including injury from direct feeding, transmitting plant diseases, and competing with vital plant symbiotic insects, leading to significant economic losses in agriculture, disruption of natural ecosystems, and environmental damage [121]. The role of NO in protecting plants against insects through regulation of various physiological and molecular processes has been reported. For example, endogenous NO was shown to activate signaling molecules and plant defense mechanisms during insect invasion [122]. NO can induce the expression of plant defense related genes, leading to synthesis of insect growth inhibitory proteins [31]. Studies have also shown that NO can activate various signaling pathways that trigger the production of defense-related hormones in plants, such as salicylic acid (SA), JA, and ethylene (ETH), which are known to play crucial roles in plant defense against insects [2,23,123]. NO can also enhance the production of secondary metabolites, such as alkaloids, flavonoids, and terpenoids, which can have insecticidal properties [124]. Interactions between plant signal transduction and insect feeding behaviors are crucial in the induction of plant immunity. During herbivore attacks, plant cell surface-localized pattern recognition receptors (PRRs) induce plant defense by recognizing plant-derived damage-associated patterns (DAMPs), microbe-associated molecular patterns (MAMPs), herbivore-associated molecular patterns (HAMPs), and phytocytokines, resulting in pattern-triggered immunity (PTI) against pathogens [23,125]. However, very few PRRS for HAMPs have been described [126]. Herbivore attacks can alter the plasma membrane potential and signaling molecules, such as ROS and RNS [125]. NO is involved in plant stress tolerance and acts as a signaling molecule during herbivore attacks [127]. Accumulation of NO and H_2_O_2_ was shown to coincide with the induction of JA, ETH, and SA hormones that sequentially appeared within the first 24–96 h after the aphid *Acyrthosiphon pisum* fed on the leaves of a pea seedling [128]. The simultaneous production of phytohormones, ROS, and RNS at the same time points suggested their synergistic defense action against aphid infection in pea plants. Moreover, application of NO donors to pea plants infested with *A. pisum* revealed the induction of deterrent defense reactions, leading to a reduced population of *A. pisum* [129]. In addition, NO has been shown to play significant roles in postharvest pest control [130].

### 5.2. Role of NO in Plant and Pathogens Interaction

Pathogens, such as viruses, bacteria, and fungi, are disease-causing microorganisms that can infect and damage plants. Pathogens can enter the plant through wounds, natural openings such as stomata, or by penetrating the plant cell walls. Once inside the host, pathogens can damage plants by releasing toxins or by competing with other plants for nutrients and resources. Pathogens can also weaken the plant defense system, making it more vulnerable to other biotic and abiotic stressors [131,132]. The roles of NO in the protection of plants from fungi, viruses, and bacteria have been well characterized [10], and some are discussed below.

#### 5.2.1. Antiviral Effects of NO

Plants are exposed to a vast array of viruses. In response to tomato mottles mosaic virus, a significant increase of NO, along with phytohormones, was observed in tomato plants [133]. Rice plants infected with black-streaked dwarf virus (RBSDV) exhibited increased production of NO, while application of NO donors further improved the defense system of rice plants against RBSDV infection [134]. A rice *Osnia2* mutant with decreased NO levels compared to the wild type plants during response to RBSDV infection showed recovery in NO accumulation after treatment with NO donors; moreover, the study demonstrated that NO treatment could increase the production of SA and expression of stress-related genes, such as *OsICS1*, *OsPR1b*, and *OsWRKY45*. However, treatment of NO inhibitor decreased the tolerance of rice to RSV infection. The study also observed an increase in the expression of stress-related genes, such as *OsPR1b* and *OsWRKY45*. Application of brassinosteroids (BRs) was previously shown to enhance Arabidopsis tolerance to viral infections by increasing NO production [135]. In contrast, application of cPTIO or NR inhibitor (tungstate), which are NO scavengers, could reverse its beneficial effects and increase plant susceptibility to viral infections. NO has also been shown to enhance tolerance to various viral infections in numerous plants [136,137], which suggests the potential of NO donors in increasing plants survival under different viral diseases.

#### 5.2.2. Antibacterial Effects of NO

NO has been reported to regulate plant defense system against pathogenic bacterial infection by triggering a cascade of defense responses that inhibit growth and the spread of pathogens [10]. NO can induce the expression of defense-related genes and activate the production of bactericidal ROS [23]. NO can also regulate the activity of plant defense proteins, such as mitogen-activated protein kinases (MAPKs), which play crucial roles in the regulation of stress-related gene expression levels [138]. NO can induce the production of stress-related hormones, such as SA, JA, and ETH, which crucially regulate the plant defense responses to pathogenic bacterial infections [12,26]. Various NO-induced genes, including *CLV1*, *CLV3*, and *ILL6*, were shown to positively regulate the Arabidopsis defense system, such as plant basal defense, a resistance mediated by resistance gene (*R*-gene-mediate resistance) and SAR to enhance tolerance against virulent and avirulent *Pseudomonas syringae* pv. *Tomato* (*Pst*) DC3000 strains [2,24,139]. A previous report showed that the plant basal defense was positively regulated to protect against virulent bacterial strain *Pst* DC3000 (*Pst* DC3000 *vir*) by significantly decreasing the expression of the Arabidopsis pathogenesis-related genes (*AtPR1* and *AtPR2*) in the mutant *atclv1*, *atclv2*, and *atill6* lines compared to control plants. The study also showed that NO-induced genes could positively regulate the *R*-gene-mediated resistance in response to the avirulent *Pst* DC3000 (*Pst* DC3000 *avr*B) bacterial strain by markedly decreasing the expression of Arabidopsis *PR1* and *PR2* genes, leading to significantly increased electrolyte leakage in *atclv1*, *atclv2*, and *atill6* mutant lines compared to control plants (ref). In addition, the study also found that the NO-induced genes could positively regulate SAR in the Arabidopsis leaves in response to the avirulent *Pst* DC3000 *avr*B bacterial strain. Significant decreases in the expression of the Arabidopsis SAR pathway related genes, such as *PR1*, *PR2*, glycerol-3-phosphate dehydrogenase (*AtG3Pdh*), and azelaic inducer 1 (*AtAZI*), were also observed in the *atclv1*, *atclv2*, and *atill6* mutant lines compared to the control plants. In contrast, the Arabidopsis NO-induced genes, such as *AO3*, *NCED3*, *bZIP62*, and *DUF569*, which are associated with ABA pathway or involved in drought stress responses, had negatively regulated plant basal defense, *R*-gene mediated resistance, and SAR, but had positively regulated drought stress tolerance [16,17,139,140]. Arabidopsis inoculation with the virulent and avirulent *Pst* DC3000 strains led to the identification of candidate genes in the resistant phenotype, which showed significantly decreased bacterial growth and EL accompanied with increased expression of *PR1*, *PR2*, *G3DPH*, and *AZI* genes. Under drought stress, a susceptible Arabidopsis mutant phenotype with a significantly decreased ABA production, survival percentage, and EL, as well as significantly decreased expression of *ABA2*, *ABA3*, *ABI2*, *DREB1*, *DREB2*, *APX1*, and *NCED3* genes was observed. Shi, et al. [141] detected an increase in the endogenous level of NO after MEL application, which indicated that MEL could induce NO production and other mechanisms associated with innate immunity during bacterial infection in Arabidopsis. In addition, application of MEL and NO donor was shown to increase Arabidopsis resistance to *Pst* DC3000 bacterial strain, while the application of NO scavenger declined the endogenous NO levels, which increased plant susceptibility to *Pst* DC3000 bacterial strain. Moreover, treatment of tomato plants with the NO donor, SNP, enhanced the expression of defense-related genes and reduced bacterial infection severity [142]. Overall, the role of NO in the response to plant pathogenic bacteria is an active research area, and further studies are needed to fully elucidate the molecular mechanisms involved in their interactions.

#### 5.2.3. Antifungal Effects of NO

**Figure 3:**
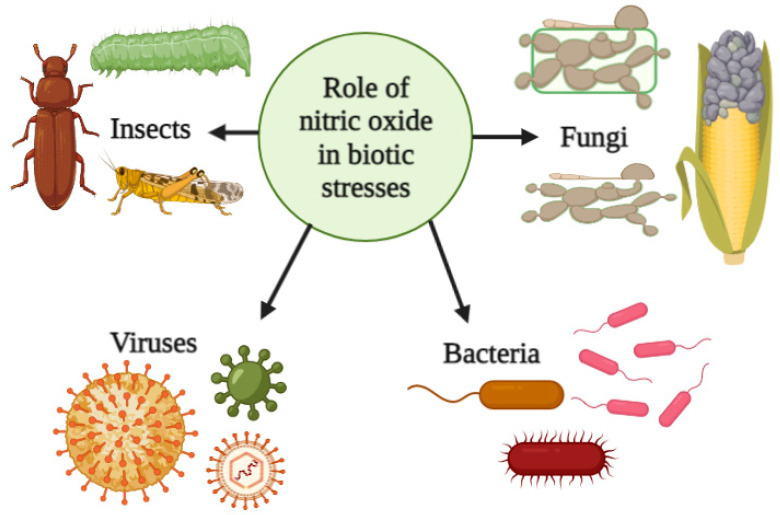
Schematic illustration of NO activities during biotic stress. This figure was generated with BioRender.com.

Pathogenic fungi can infect plants through numerous routes and spread disease. Necrotrophic fungal infections frequently exhibit a wide host range and kill and consume nutrients released from the dead tissues of their hosts, while biotrophic fungal diseases do not discharge poisonous substances and exhibit host specificity; they frequently secrete effectors to inhibit the host immune system. Hemibiotrophic fungal pathogens initially thrive as biotrophs before transitioning to necrotrophs, which is an intermediate life stage between the necrotrophic and biotrophic forms [143]. The necrotrophic/biotrophic properties of fungal pathogens control the concentration and the spatiotemporal patterns of NO in plant tissues, which impact its specific roles during pathogenic fungal infection. Interestingly, fungal organisms are potentially associated with NO generation and metabolism during plant–fungal pathogen interactions [144]. Application of SNP significantly halted lesion development of apple fruit inoculated with *Penicillium expansum* [145]. Similarly, NO has been shown to protect plants against other fungi, such as *Fusarium oxysporum* [146] and *Alternaria alternata* [147]. Similarly, NO has been shown to enhance the resistance of barley, *Arabidopsis*, and wheat against biotrophic fungi, such as *Blumeria graminis*, *Golovinomyces orontii*, *Erysiphe pisi*, and *Puccinia triticina* [143]. Overall, the application of NO and NO donors can enhance the expression of stress-related genes and increase the production of stress-related proteins and hormones, such as SA and JA [148]. The roles of NO in the alleviation of herbivorous insects, fungi, virus, and bacterial infections are illustrated in Figure 3.

## 6. Conclusions and Future Trajectories

NO is a versatile signaling molecule that is essential for plant growth and development, as well as plant responses to abiotic and biotic stressors. NO is synthesized in plants through various routes, such as oxidative, reductive, and nonenzymatic pathways. NO has been reported to alleviate the adverse effects of environmental challenges on plants via the regulation of various physical, biochemical, and molecular plant processes under different environmental conditions. Under abiotic stresses, NO can activate the antioxidant system to minimize oxidative damage. Additionally, NO can regulate stomatal closure, improve water uptake, and promote root growth, all of which are crucial adaptive strategies in plants subjected to stress. Additionally, NO can regulate plant immune responses, such as the production of ROS, the expression of defense-related genes, and the production of phytohormones under biotic stress. Moreover, NO can induce SAR, which prepares the plant to respond more efficiently to future pathogen attacks. Understanding the mechanisms underlying the regulation of NO in plants can provide valuable insights into developing strategies for improving crop productivity and enhancing plant resistance to abiotic and biotic stressors. Based on the previous investigations, various NO-donors could be used for the growth and development of plants, even under stressful conditions. Such a critical role of NO compelled researchers to identify more suitable NO-donors for large scale agricultural practices to promote the productivity of crop plants.

## Figures and Tables

**Table 1:** The roles of NO in the mitigation of different abiotic stresses in plants.

Plant Species	Stress	NO Donor	NO Function	References
Maize	Drought	100 µM SNP	Regulation of water status	[107]
Mustard	Drought	100 µM SNP	Antioxidant system activation	[103]
Soybean	Drought	100 µM SNP	Stimulation of antioxidant system and osmotic adjustment	[61]
Soybean	Flooding	100 µM SNP	Modulation of growth and physio-molecular responses	[18]
Tomato	Flooding	500 µM SNP	Induction of flooding stress related genes	[108]
Rice	Chilling	100 µM SNP	Regulation of water balance and antioxidant system	[76]
Melon	Chilling	200 mM SNP	Regulation of gene expression	[109]
Tea	Chilling	500 µM/L SNP	Stimulation of antioxidant system	[110]
Rice	Heat	100 µM SNP	Protective effects on photosynthesis	[75]
Tomato	Heat	100 µM SNP	Enhancement of antioxidant system and alleviation of oxidative stress markers	[106]
Wheat	Heat	100 µM SNP	Regulation of osmolytes and antioxidants	[111]
*Vigna radiata*	Salinity	0.06 mM SNP	Modulation of oxidative stress markers and antioxidant system	[89]
Lentil	Salinity	100 µM SNP	Modulation of plant growth and biochemical properties	[112]
Wheat	Salinity	5 mM SNP	Alleviation of oxidative stress	[113]
Rice	Chromium	100 µM NaHS75 µM SNP	Regulation of antioxidant system	[114]
Lupin	Nickle	0.4/0.6 mM SNP	Modulation of antioxidant system	[115]
Wheat	Cadmium	0.10 mM SNP	Reduction in oxidative stress markers	[116]

## Data Availability

Not applicable.
